# Profiling of the Tetraspanin CD151 Web and Conspiracy of CD151/Integrin β1 Complex in the Progression of Hepatocellular Carcinoma

**DOI:** 10.1371/journal.pone.0024901

**Published:** 2011-09-22

**Authors:** Ranjan Prasad Devbhandari, Guo-Ming Shi, Ai-Wu Ke, Fei-Zhen Wu, Xiao-Yong Huang, Xiao-Ying Wang, Ying-Hong Shi, Zhen-Bin Ding, Yang Xu, Zhi Dai, Jia Fan, Jian Zhou

**Affiliations:** 1 Liver Cancer Institute, Shanghai Medical School, Fudan University, Zhongshan Hospital, Shanghai, People's Republic of China; 2 Department of Gastroenterology, Tongji University School of Medicine, Shanghai Tenth People's Hospital, Shanghai, People's Republic of China; 3 Laboratory of Epigenetics, Institutes of Biomedical Sciences, Fudan University, Shanghai, People's Republic of China; 4 Cancer Center, Institutes of Biomedical Sciences, Fudan University, Shanghai, People's Republic of China; National University of Singapore, Singapore

## Abstract

Tetraspanin CD151 has been implicated in metastasis through forming complexes with different molecular partners. In this study, we mapped tetraspanin web proteins centered on CD151, in order to explore the role of CD151 complexes in the progression of hepatocellular carcinoma (HCC). Immunoprecipitation was used to isolate tetraspanin complexes from HCCLM3 cells using a CD151 antibody, and associated proteins were identified by mass spectrometry. The interaction of CD151 and its molecular partners, and their roles in invasiveness and metastasis of HCC cells were assayed through disruption of the CD151 network. Finally, the clinical implication of CD151 complexes in HCC patients was also examined. In this study, we identified 58 proteins, characterized the tetraspanin CD151 web, and chose integrin β1 as a main partner to further investigate. When the CD151/integrin β1 complex in HCC cells was disrupted, migration, invasiveness, secretion of matrix metalloproteinase 9, and metastasis were markedly influenced. However, both CD151 and integrin β1 expression were untouched. HCC patients with high expression of CD151/integrin β1 complex had the poorest prognosis of the whole cohort of patients. Together, our data show that CD151 acts as an important player in the progression of HCC in an integrin β1-dependent manner.

## Introduction

The dismal prognosis of hepatocellular carcinoma (HCC) is mostly attributed to high metastasis. This disease has attracted the considerable attention of investigators from the bench to the clinic [1], [2]. Metastasis is a multi-step cascade involving migration of tumor cells from the original site, evasion of host defense systems, subsequent location to distant organs, and growth of secondary tumors [3], [4]. Throughout the cascade, from its original location to distant sites, there is constant interplay between metastasizing tumor cells and the changing microenvironment, leading to the breakdown of normal cell adhesion which facilitates cell migration [5].

Integrins, which are composed of a non-covalent heterodimer of α- and β-subunits, mediate signals from the extracellular matrix (ECM) that help to regulate cell migration, differentiation, cell cycle progression, apoptosis, phagocytosis, ECM assembly, and metalloproteinase activity [5]. Within the integrin family, integrin β1 has been widely studied in the biology of solid tumors, as its expression positively correlates with poor prognosis in a several malignancies, including breast cancer [6] and HCC [7]. These effects are mostly attributed to the binding of integrin β1 to ligands, fibronectin, and laminin, as well as the modulation of cytoskeleton-signaling associated with cell migration, ECM assembly, and metalloproteinase activity [5]. However, our previous study showed that high levels of integrin β1 expression in HepG2 cells with low metastatic potential and a high percentage of integrin β1- positive cells in most HCC samples [8]. These results indicate that the metastatic potential of HCC cells is not completely dependent on integrin β1 expression.

Tetraspanins (TM4SF) possess four transmembrane domains: a small intracellular loop, intracellular N- and C-termini with short cytoplasmic tails, and two extracellular loops, the larger of which contains a distinctive pattern of cysteine residues that helps to define the family [9]. The most striking feature of tetraspanin molecules is that they can assemble multimolecular signaling complexes at the cell surface such as integrins, membrane receptors, intracellular signaling molecules, other tetraspanins, and immunoglobulin superfamily proteins at tetraspanin-enriched microdomains (TEMs) to form “a tetraspanin web” [10], [11]. This “tetraspanin web” can thus serve as molecular facilitators or organizers of multimolecular complexes on the cell surface [9], [12], [13]. Recent studies have demonstrated that the great heterogeneity in the composition of the “tetraspanin web”, and the dynamics of tetraspanin complexes, confer great flexibility to tetraspanins that allow for specificity and functional differences [14]. For example, CD82 can form complexes with the GM2/GM3 heterodimer and inhibit cell motility through the CD82-c-Met or integrin-c-Met pathway [15]. CD82 attenuates α6 integrin-mediated cellular morphogenesis through modulation of integrin α6 signaling and internalization of integrin α6 [16]. Hence, to uncover the functions of tetraspanin proteins, it is critical to identify laterally interacting partner proteins [12], [13].

Tetraspanin CD151 (also named as SFA-1 and PETA-3), is an important member of the tetraspanin family, and has been widely studied in several malignant tumors [13], [17], [18], [19]. Evidence has demonstrated that CD151 forms complexes with integrins, c-Met, other tetraspanins, and itself, and is implicated in pathological processes associated with cancer progression [12], [20], [21]. For example, modified HeLa cells overexpressing CD151 have more migratory abilities than control cells expressing little CD151 [22]. Other reports have shown that antibodies against CD151 can inhibit cell migration [23]. Previously, we determined that CD151 was a crucial molecule involved in tumor cell invasion [18], tumor neo-angiogenesis [19], and epithelial-mesenchymal transition (EMT) [8]. We also showed that it was associated with the progression and metastasis of HCC by forming complexes with c-Met or integrin α6. This suggests that CD151 is likely to participate in metastasis through forming complexes with different molecular partners, which contributes to heterogeneous characteristics in terms of the aggressive potential and prognosis of HCC [24]. However, the role of CD151 in malignant tumors is not simple. Overexpression of CD151 significantly increases intercellular adhesiveness and delays wound closure in a scratch assays [25], [26]. Conversely, downregulation of CD151 expression promotes invasiveness of breast cancer cells [27]. Thus, CD151 is likely to possess diverse and unpredictable functions in different cellular environments. Thus, the “tetraspanin web” centered on CD151, and its interaction in HCC remains to be investigated.

A more thorough description of the composition and organization of the tetraspanin web, and deeper exploration into the interaction of tetraspanin complexes, are of importance in understanding the function of these molecules. In the present study, we performed proteomic analysis of CD151-containing complexes by mass spectrometry (MS), and then evaluated the interaction of CD151 and integrin β1 *in vivo* and *in vitro*. Finally, we assayed the roles of CD151/β1-integrin complexes in the prognosis of HCC patients.

## Results

### Characterization of the tetraspanin CD151 network in HCC cells

**Figure 1:**
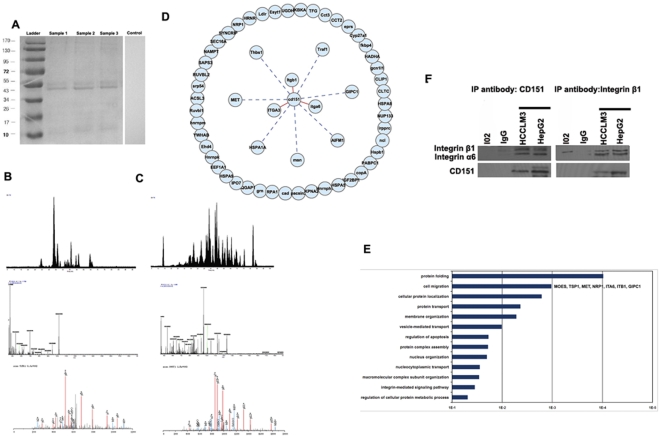
Identification of tetraspanin CD151 network and functional analysis. CD151-containing complexes were solubilized using the mild detergent, Brij97, and were isolated by immunoprecipitation using specific CD151 mAbs. Associated proteins were eluted using Triton X-100 and separated by SDS-PAGE (A). After in-gel trypsin digestion of proteins, the resulting peptides were analyzed using LC-MS/MS (B and C). The peptides were separated by nano-HPLC. Total ion count was measured and visualized on a chromatogram (upper panel). At a precise time (e.g. 55.33 min for CD151 and 67.18 min for integrin β1, dotted straight line), the mass spectrum obtained is shown (middle panel) in which a parent ion can be selected (e.g. m/z = 643.3489 for CD151 and 1010.0838 for integrin β1, black arrow). Fragmentation of that parent ion led to MS/MS spectrum generation containing b and y ions, and thus sequence information of the parent ion (lower panel). The amino acid sequence can be deduced after searches in the NCBI database using the program SEQUEST. The putative sequence of the peptide is shown with associated Xcorr and ΔCn. This peptide sequence led to the identification of CD151 and integrin β1. These peptides were converted into a gene symbol and rearranged according to GO functional enrichment provided by DAVID website. Then, the “tetraspanin CD151 web” was organized by the cytoscape (Ver: 2.6.2, http://www.cytoscape.org/), searching target genes that interacted with CD151, and removing genes that were not contained in our target genes using a data source (NCBI Entrez EUtilities Web Service Client) (D). Gene ontology analysis of the 57 molecular partners was performed. They were classified as 13 categories of biological processes including protein folding and cell migration (MOES, TSP1, c-MET, NRP1, ITA6, ITB1 and GIPC1) according to categories of the “GO Biological Process” (E). Western blotting identified CD151 formed complexes with integrin α6 and β1 in HCCLM3 cells as well as HepG2 cells, but not in normal hepatocytes L-02 (F).

With the knowledge of the structure and functional patterns of tetraspanins, we mapped tetraspanin complexes centered on CD151, to address the interaction of CD151 with its molecular partner in HCC cells in the context of other regulatory mechanisms. In present study, we chose HCCLM3 cells to do the immuoprecipitation because HCCLM3 cells have the highest expression of CD151 protein based on our previous study [18] . After isolation of CD151-containing complexes by immunoprecipitation, proteins were eluted using the stringent detergent, Triton X-100, which dissociates tetraspanin-tetraspanin associations [28]. Proteins were then resolved by SDS-PAGE (Fig. 1A), and CD151-containing complexes were identified by mass spectrometry. A common set of 58 proteins was obtained when the significance threshold was set at p<0.05 (Table 1); identification of CD151 and integrin β1 are shown in Fig. 1B and 1C. Bioinformatic analysis of the enriched partners in various categories displayed an integrated tetraspanin network centered on CD151 in HCC cells. We found that 10 of the 57 CD151-associated partner proteins identified, were previously reported to directly or indirectly associate with CD151. Based on the type of interaction, we classified three levels of interactions in the tetraspanin network (Fig. 1D). As shown in Fig. 1D, CD151 formed the first level of complexes with tetraspanin partners integrin α3, α6, and β1 all of which were dependent on direct interactions. We also observed seven functional complexes at the second level, which indirectly interacted with CD151 (Fig. 1D). Interestingly, 47 novel molecules were identified, which unknown interacted with CD151 (Fig. 1D).

**Table 1:** List of identified 58 proteins.

Protein name	Score	Protein uniprot accession no.	Theoretical molecular mass(Da)	No. of peptides
Integrin β1	1037	P05556	88407	12
GRP-75	886	P38646	73635	16
Heat shock 70 kDa protein 8	560	P11142	70854	14
Integrin α3	542	P26006	118622	9
Cell proliferation-inducing gene 32 protein	497	P07814	170540	12
GRP-78	409	P11021	72288	9
p195	168	P46940	189134	4
HSP70-1/HSP70-2	168	P08107	70009	4
TP-alpha	156	P40939	82947	4
NAmPRTase	149	P43490	55487	4
CD151	128	P48509	28295	2
LDL receptor	114	P01130	95314	1
EF-1-alpha-1	109	P68104	50109	1
RuvB-like 2	106	Q9Y230	51125	3
Moesin	103	P26038	67778	2
UDPGDH	101	O60701	54989	2
PABP-1	100	P11940	70626	5
SEC16 homolog A	99	O15027	233373	2
RuvB-like 1	97	Q9Y265	50196	2
Neuropilin-1	90	O14786	103055	1
Integrin α6	88	P23229	126539	2
hnRNP Q	88	O60506	69560	2
Granulins	87	P28799	63500	1
Alpha-coat protein	87	P53621	138244	2
TIP-2	86	O14908	36027	1
CAD protein	86	P27708	242829	1
Restin	83	P30622	160891	1
PACSIN2	81	Q9UNF0	55704	4
LRP 130	81	P42704	157805	2
TCP-1-beta	77	P78371	57452	1
RP-A p70	70	P27694	68095	2
Nucleolin	66	P19338	76568	1
Cytochrome P450 27	63	Q02318	60196	1
Thrombospondin-1	61	P07996	129300	2
TCP-1-gamma	53	P49368	60495	1
HGF receptor	52	P08581	155427	2
HspB1	51	P04792	22768	1
Protein HS1	50	P27348	27764	3
TNFαR1	48	P50555	50696	2
Protein TFG	46	Q92734	43407	1
hnRNP K	45	P61978	50944	1
PAST homolog 4	44	Q9H223	61137	1
Importin-7	43	O95373	119440	2
LACS 3	43	O95573	80368	1
IMP-1	40	Q9NZI8	63417	1
Programmed cell death protein 8	40	O95831	66859	1
hnRNP M	38	P52272	77464	1
ELP1	37	O95163	150096	1
SRP54	36	P61011	55668	1
Importin subunit alpha-2	34	P52292	57826	1
hnRNP H	33	P31943	49198	1
Extended-synaptotagmin-1	32	Q9BSJ8	122780	1
Hornerin	32	Q86YZ3	282228	1
SAPS domain family member 3	31	Q5H9R7	97608	1
Clathrin heavy chain 1	31	Q00610	191493	1
Nucleoporin Nup133	31	Q8WUM0	128898	1
p59	30	Q02790	51772	1
HsGCN1	30	Q92616	292558	1

To analyze the role of molecular partners in the tetraspanin network centered on CD151 in HCC cells, we performed gene ontology (GO) analysis of the 57 molecular partner candidates. They were classified according to categories of the “GO Biological Process” as shown in Fig. 1E. Results revealed that molecular partners included proteins involved in protein folding (HSP7C, TCPB, RUVB2, COPA, TCPG, FKBP4 and GRP75), cell migration (MOES, TSP1, c-MET, NRP1, ITA6, ITB1 and GIPC1), cellular protein localization (IMA2, 1433B, SRP54, COPA, IPO7, GRP75, CLH1 and GIPC1), protein transport (IMA2, NU133, 1433B, SRP54, SC16A, COPA, IPO7, GRP75, CLH1 and GIPC1), membrane organization (EHD4, HSP7C, TSP1, LDLR, PACN2, COPA, CLH1), vesicle-mediated transport (EHD4, HSP7C, TSP1, LDLR, PACN2, SC16A, COPA and CLH1), regulation of apoptosis (TSP1, 1433B, AIFM1, HSP71, GRP78, TRAF1, GRP75 and HSPB1), protein complex assembly (SYEP, 1433B, SRP54, TRAF1, IPO7, FKBP4 and ELP1), nucleus organization (NU133 and AIFM1), nucleocytoplasmic transport (IMA2, NU133, IPO7 and GRP75), macromolecular complex subunit organization (SYEP, NU133, 1433B, SRP54, TRAF1, IPO7, FKBP4 and ELP1), integrin-mediated signaling pathway (ITA3, ITA6 and ITB1) and regulation of cellular protein metabolic processes (TSP1, GCN1L, IF2B1, 1433B, HSPB1 and GIPC1). Among them, proteins related to protein folding and cell migration represented the most abundant groups of proteins (Fig. 1E), demonstrating that CD151 forms functional complexes with these molecular partners to participate in biological processes of HCC, including metastasis.

Consistent with previous results [8], we identified integrin α6 and β1 as molecular partners for CD151 in HCCLM3 cells and HepG2 cells as well, but not in normal hepatocytes L-02 expressing little CD151 (Fig. 1F). To further explore the function and interaction between CD151 and its partners in HCC cells, we chose integrin β1 as a core partner for the follow reasons: (i) among 57 tetraspanin partners, integrin β1 had the highest Mascot score (Table 1), (ii) analysis of the “GO Biological Process” for mapping of the tetraspanin network, showed that proteins associated with cell migration were the second most abundant category of tetraspanin partners, of which integrin β1 played a crucial role in cell migration, (iii) it is well documented that integrin β1 is tightly associated with the progression of malignant tumors, (iv) among 5 integrin subunits, integrin β1 expressed at high level in HCCLM3 cells (Fig. S1). However, metastasis of HCC is not completely dependent on integrin β1 expression, as discussed in the ‘Introduction’. Therefore, we investigated the interaction of the CD151/integrin β1 complex, and its role in invasion and metastasis.

### Location and expression of CD151/integrin β1 complexes in HCC cells

**Figure 2:**
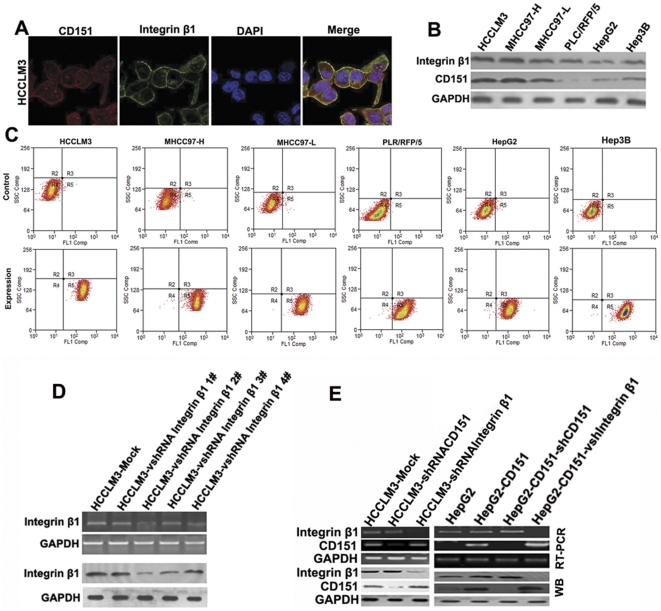
Location and expression of CD151/integrin β1 complex in HCC cells. An immunofluorescence assay was performed. CD151 was stained with red and integrin β1 was stain with green. CD151 and integrin β1 co-localized on the plasma membrane in HCCLM3 cells (A). Western blotting revealed that the expression of CD151 in high metastatic HCCLM3 cells was highest and non-metastatic HepG2 cells presented the lowest levels of CD151 among six HCC cells. The results also demonstrated high expression of integrin β1 in six HCC cells lines (B). Flow cytometry analysis showed that the high proportion of integrin β1-positive cells were in six HCC cells (C). After transfection, HCCLM3-shRNA 2# was most efficient in downregulating the expression of integrin β1 at both the protein and mRNA levels (D). The level of integrin β1 expression remained unchanged in HCC interfered with CD151 shRNA, and vice versa (E).

An immunofluorescence assay showed that CD151 and integrin β1 were co-localized on the plasma membrane in HCCLM3 cells (Fig. 2A). In line with our previous reports [8], the protein expression of CD151 in HCC cells positively correlated to its metastatic potential (Fig. 2B). Interestingly, all HCC cells used in this study had high protein expression of integrin β1 (Fig. 2B). Flow cytometry showed a high proportion of integrin β1-positive cells in HCCLM3 cells, MHCC97H, MHCC97L, PLC/PRF/5, Hep3B and HepG2 cells (Fig. 2C), indicating that the metastatic potential of HCC cells is not associated with the percentage of integrin β1-positive cells. The expression of CD151 or integrin β1 in HCCLM3-shRNA was downregulated at both the protein and mRNA levels compared to parental cell lines (Fig. 2D and E). As expected, the level of integrin β1 expression remained unchanged in shRNA-CD151-HCCLM3 cells, and vice versa (Fig. 2D and E), suggesting that the expression of integrin β1 is independent of that of CD151.

### CD151 increases invasion and mobility of HCC cells in an integrin β1-dependent manner

**Figure 3:**
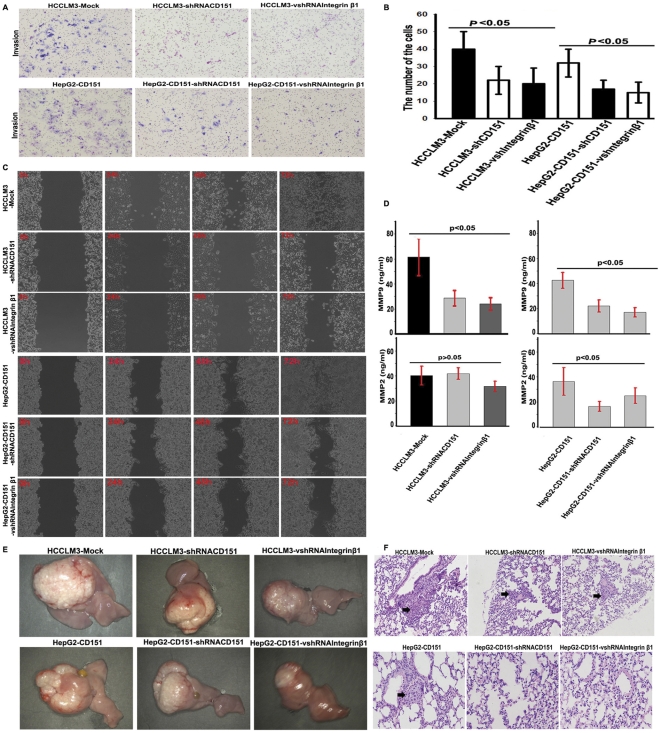
CD151 increased invasion, mobility and metastasis of HCC cells in an integrin β1-dependent manner. Transwell assays showed that the number of invasive HCCLM3-mock and HepG2-CD151 cells were significantly higher than those of shRNA-CD151-HCCLM3, shRNA-integrin β1-HCCLM3 cells and HepG2-CD151-shRNA-CD151, HepG2-CD151-shRNA-integrin β1, respectively (A and B). Wound healing assays revealed an apparent decrease in the mobility of HCCLM3 cells transfected with shRNA-CD151 or shRNA-integrin β1. Similarly, disruption of CD151 or integrin β1 in HepG2-CD151 cells, HepG2-CD151-shRNA-CD151 or shRNA-integrin β1 showed the impaired mobility (C). ELISA showed that the concentration of MMP9 in the supernatant from HCCLM3-mock and HepG2-CD151 cells was significantly higher than that of shRNA-CD151-HCCLM3 cells, shRNA-integrin β1-HCCLM3 cells and HepG2-CD151-shRNA-CD151 or shRNA-integrin β1, respectively (D). However, no significant changes of MMP2 were observed in HCC cells after interference with CD151 or integrin β1 (D). After construction of metastasis model in situ, tumor volumes of HCCLM3-mock-derived xenografts were significantly larger than those of HCCLM3-shRNA-CD151 and HCCLM3-shRNA-integrin β1 (E). The tumor volumes of CD151-HepG2-derived xenografts were significantly larger than those of HepG2-CD151-shRNA-CD151 and HepG2-CD151-shRNA-integrin β1 (E). Serial sections showed that the pulmonary metastasis rates and metastatic tumor clusters in the HCCLM3-mock group were higher than that of HCCLM3-shRNA-CD151 group and the HCCLM3-shRNA-integrin β1 group. The pulmonary metastasis rates of CD151-HepG2 were markedly higher that of HepG2-CD151-shRNA-CD151 and HepG2-CD151-vshRNA-Integrinβ1 groups (F).

Invasion assays were used to investigate the interaction of the CD151/integrin β1 complex and its role in the invasiveness of HCC cells. Results showed that the number of invaded HCCLM3-mock cells were 40±10, significantly higher than those of shRNA-CD151-HCCLM3 (22±8) and shRNA-integrin β1-HCCLM3 (20±9) cells (Fig. 3A and B). After transfection of CD151 cDNA into HepG2 cells, the number of invaded HepG2-CD151 cells was higher than that of HepG2-mock cells (32±8 *verse* 16±5). Of note, when CD151/integrin β1 expression in HepG2-CD151 cells was silenced, the number of invaded cells markedly decreased (17±5 and 15±6, Fig. 3A and B), indicating that CD151 and integrin β1 have synergistic roles in the invasiveness of HCC cells.

Cell migration assays showed an apparent decrease in the mobility of HCCLM3 cells transfected with shRNA-CD151 or shRNA-integrin β1 (Fig. 3C). However, after transfection with the CD151 plasmid, HepG2 cells had increased migratory capabilities. Representative photography indicated accelerated closure in HCCLM3-mock and HepG2-CD151 cells, compared to shRNA-CD151-HCCLM3, shRNA-integrin β1-HCCLM3, HepG2-CD151-shRNA-CD151, and HepG2-CD151-shRNA-integrin β1 cells (Fig. 3C). The aforementioned data indicate that CD151 likely increases the invasion and mobility of HCC cells in an integrin β1-dependent manner.

ELISA assays showed that the concentration of matrix metalloproteinase 9 (MMP9) in the supernatant of HCCLM3-mock cells was significantly higher than that of shRNA-CD151-HCCLM3 and shRNA-integrin β1-HCCLM3 cells (Fig. 3D). Similarly, after transfection with the CD151 plasmid, HepG2-CD151 cells showed increased secretion of MMP9. However, when HepG2-CD151 cells were interfered with shRNA-CD151 or shRNA-integrin β1, they showed decreased secretion of MMP9 (Fig. 3D). Under these conditions, no significant changes in the MMP2 expression were observed in the modified HCC cells interfered with shRNA-CD151 or shRNA-integrin β1, compared to parental cells (Fig. 3D). These data indicate that CD151 forms a complex with integrin β1 and modulates MMP9 expression and secretion.

### Interaction of proteins within the CD151/integrin β1 complex promotes HCC metastasis *in vivo*

To further investigate the interaction and role of the CD151/integrin β1 complex in metastasis, we inoculated HCC cells in liver parenchyma of nude mice. Tumor volumes of HCCLM3-mock-derived xenografts were 1.95±0.6 cm^3^, which were significantly larger than those of HCCLM3-shRNA-CD151 (0.71±0.10 cm^3^) and HCCLM3-shRNA-integrin β1 (0.59±0.06 cm^3^). Tumor volumes of CD151-HepG2-derived xenografts were 1.89±0.6 cm^3^, which were significantly larger than those of HepG2-CD151-shRNA-CD151 and HepG2-CD151-shRNA- integrin β1 (0.51±0.05 cm^3^ and 0.47±0.04 cm^3^, respectively, P<0.01; Fig. 3E). Serial sections confirmed that pulmonary metastasis rates, and metastatic tumor clusters per mouse were 100% (5/5) and 210±63 in the HCCLM3-mock group, but were 40% (2/5) and 124±55 in the HCCLM3-shRNA-CD151 group, 40% (2/5) and 164±46 in the HCCLM3-shRNA-integrin β1 group, 60% (3/5), and 145±43 in the CD151-HepG2 group. There was no lung metastasis in the HepG2-mock, HepG2-CD151-shRNA-CD151, and HepG2-CD151-vshRNA-Integrinβ1 groups (P<0.05; Fig. 3F).

### Overexpression of the CD151-integrin β1 complex correlates with poor prognosis of HCC patients

**Figure 4:**
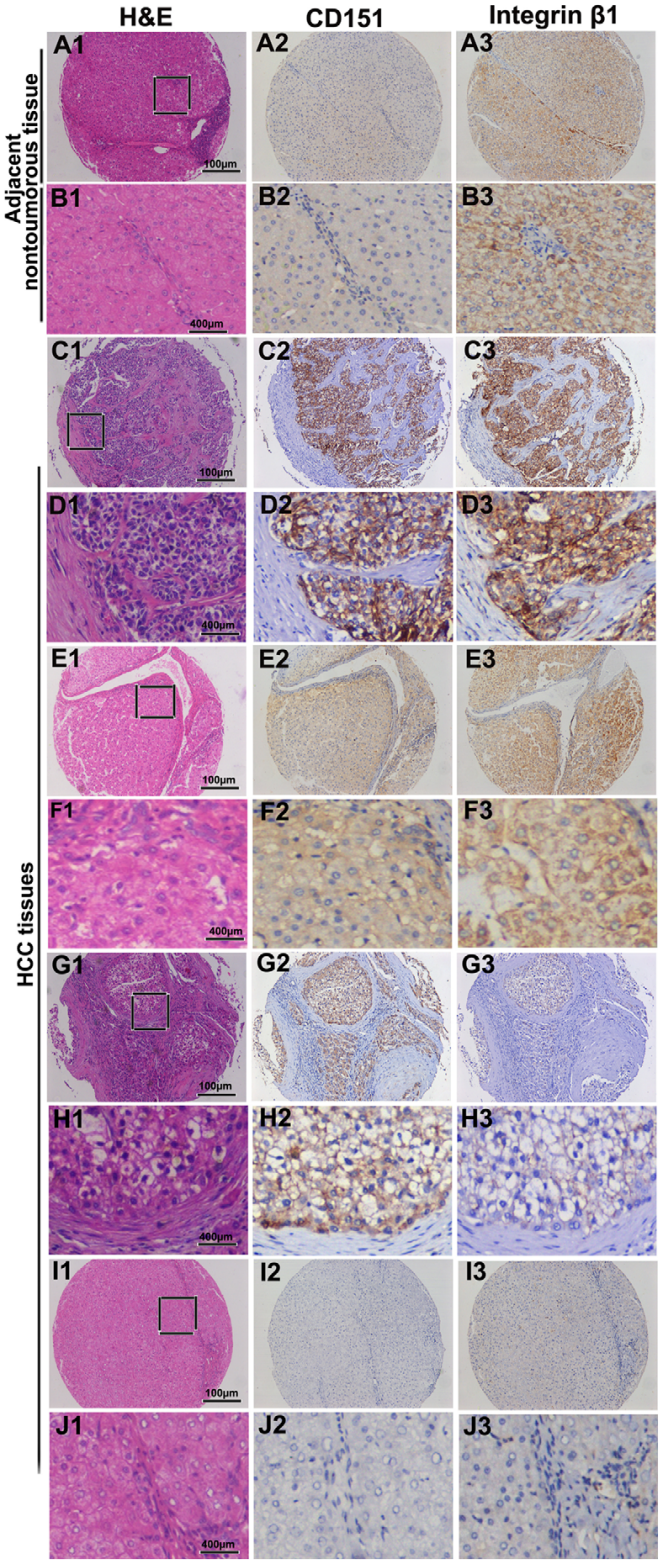
Expression of CD151/integrin β1 complex in HCC patients by immunohistochemistry. After identification by H&E staining (A1, B1, C1, D1, E1, F1, G1, H1, I1, and J1), CD151 was mostly located on the membrane of tumor cells, with comparable variation (A2, B2, C2, D2, E2, F2, G2, H2, I2, and J2) and strong integrin β1 expression in most HCC cells, stroma fibroblasts, and epithelial cells of the bile duct (A3, B3, C3, D3, E3, F3, G3, H3, I3, and J3). A and B refer to adjacent nontumoral tissue. Representative tumor tissues are shown :C and D high expression of CD151 and integrin β1, E and F high expression of CD151 and low expression of integrin β1, G and H high expression of integrin β1 and low expression of CD151, I and J low expression of CD151 and integrin β1.

**Figure 5:**
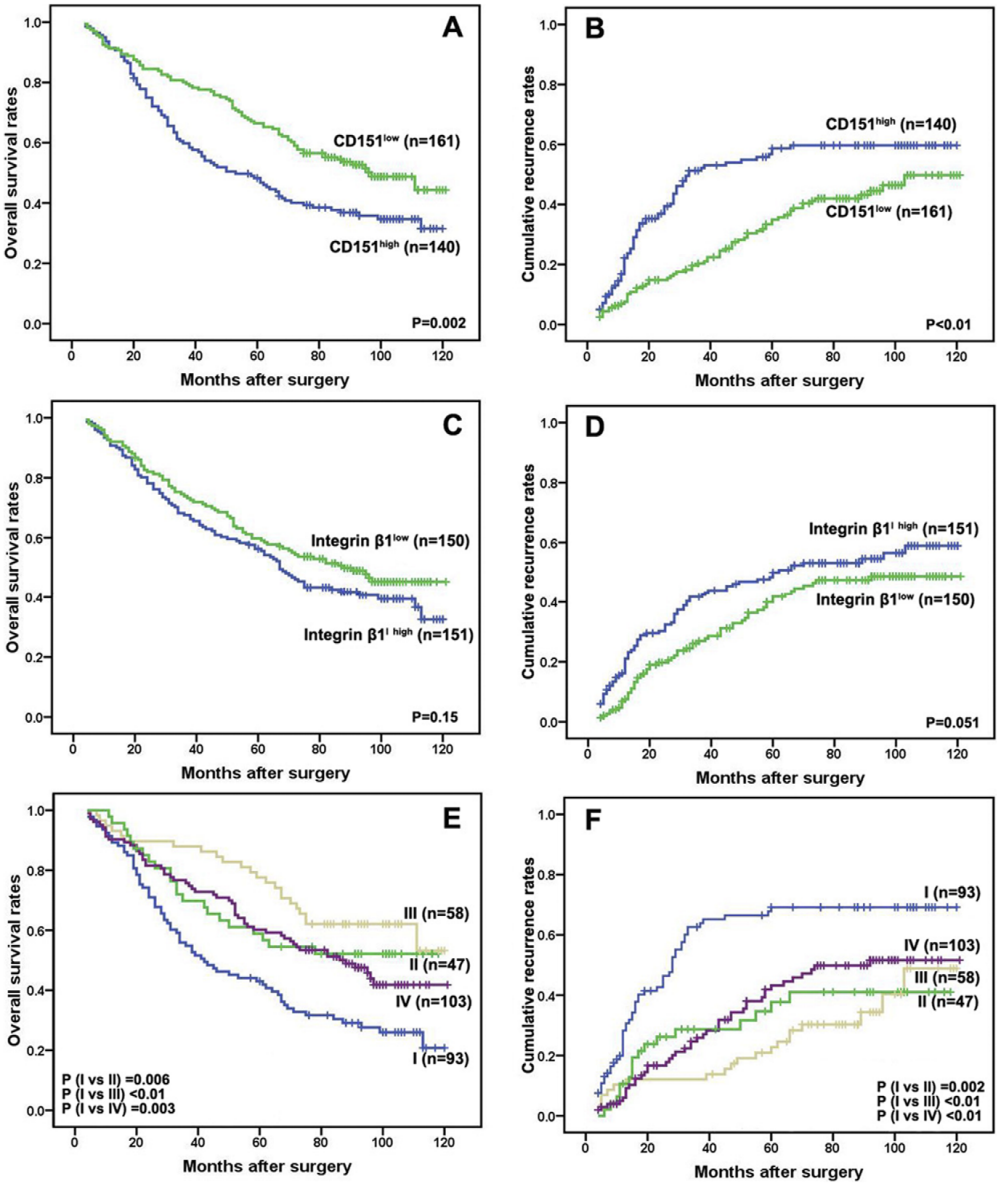
Prognostic roles by Kaplan-Meier analysis. Kaplan-Meier analysis revealed that patients with high CD151 expression had poor prognosis in terms of overall survival (A) and recurrence rate (B). However, no difference in overall survival (C) and recurrence rate (D) was observed between high integrin β1 expression group and low expression group. Patients were classified into four groups according to their CD151 and integrin β1 density: group I, high expression of both markers; group II, high expression of CD151 alone; group III, high expression of integrin β1 alone and group IV, low expression of both markers. Kaplan-Meier analysis revealed that HCC patients with overexpression of CD151/ integrin β1 presented with the worst overall survival (E) and the highest cumulative recurrence rate (F).

Here, we aimed to investigate the role of the CD151/integrin β1 complex in a larger series of HCC patients. To this end, we used immunohistochemistry to determine the expression of the CD151/integrin β1 complex in consecutive tissue microarray (TMA) slides consisting of 301 HCC and adjacent notumorous samples (Fig. 4A–J). As reported in our previous study, CD151 was mostly located on the membrane of tumor cells [8]. Strong positive integrin β1 expression was observed in most HCC cells, as well as the stroma fibroblasts and epithelial cells of the bile duct. Integrin β1 expression in tumor cells was localized in the cytoplasm, and had a diffuse or granular pattern (Fig. 4A–J). We then analyzed the relationship between expression of the CD151/integrin β1 complex and clinicopathological features of HCC. HCC characterized by microvascular invasion, large tumor (>5 cm) size, and high TNM stage, were prone to higher expression of the CD151/integrin β1 complex (Table 2). Kaplan-Meier analysis revealed that patients with high levels of CD151 expression had a poorer prognosis in terms of shorter OS and higher recurrence rates, compared to those with low expression (Fig. 5A and B). Expectedly, integrin β1 expression alone did not correlate to prognosis in this cohort of patients (Fig. 5C and D). Interestingly, patients were classified into four sub-groups according to their CD151 and integrin β1 density. The sub-group with high expression of the CD151/integrin β1 complex had the poorest prognosis of the whole cohort of patients (Fig. 5E and F). Univariate analysis revealed that CD151, microvascular invasion, and TNM showed prognostic significance, with poor prognosis for both OS and recurrence (Table 3). However, integrin β1 expression alone was not related to OS or recurrence. Multivariate analysis showed that CD151 and CD151/integrin β1 complex expression were independent predictors for OS or recurrence (Table 3). The aforementioned data indicates that CD151 functions as a molecular facilitator in the progression of HCC in an integrin β1-dependent manner.

**Table 2:** Correlations of CD151 and integrin β1 expression with clinicopathological features of 301 HCCs.

variables	CD151 staining		*P* value	Integrinβ1 staining		*P* value
	high	low		high	low	
Sex						
male	116	137	0.597	128	125	0.734
female	24	24		23	25	
Age, year						
≥52	73	80	0.671	83	70	0.15
<52	67	81		68	80	
HBsAg						
positive	115	130	0.756	123	122	0.978
negative	25	31		28	28	
Preoperative treatment						
yes	63	62	0.254	67	58	0.315
no	77	99		84	92	
Child -Pugh score*						
A	140	160	1	150	150	1
B	0	1		1	0	
Serum AFP, ng/ml						
≤20	64	82	0.366	81	65	0.074
>20	76	79		70	85	
Tumor number						
single	115	145	0.046	129	131	0.63
multiple	25	16		22	19	
Microvascular invasion						
yes	16	6	0.01	16	6	0.028
none	125	155		135	144	
Tumor encapsulation						
complete	55	54	0.301	50	59	0.262
none	85	107		101	91	
Tumor differentiation						
I/II	39	50	0.544	42	47	0.504
III/IV	101	111		109	103	
Tumor size (cm)						
≤5	71	102	0.027	78	95	0.04
>5	69	59		73	55	
TNM stage						
I/II	39	21	0.001	37	23	0.046
III	101	140		114	127	

Abbreviations and Note: HCC, hepatocellular carcinoma; AFP, alpha-fetoprotein; TNM, tumor node metastasis; HBsAg, hepatitis B surface antigen; NS, not significant; The X^2^ test was used for comparison between groups. 50% for CD151, Median of intensity for integrin β1 were used as the cut-off value.

*Fisher's exact test.

**Table 3:** Univariate and multivariate analyses of factors associated with survival and recurrence.

factor	Overall survival				Cumulative Recurrence			
	Univariate	Multivariate			Univariate	Multivariate		
	P	HR	95% CI	P	P	HR	95% CI	P
Sex (male versus female)	0.835			NA	0.487			NA
Age (<52 versus >52 years)	0.234			NA	0.641			NA
HBsAg (negative versus positive)	0.563			NA	0.215			NA
Child-Pugh score (A versus B)	0.637			NA	0.626			NA
Serum AFP (>20 versus <20 ng/mL)	0.037	1.335	0.978–1.822	NS	0.379			NA
Tumor size (>5 versus <5 cm)	0.002	1.378	1.006–1.887	0.046	0.114			NA
Tumor number (multiple versus single)	0.003	1.637	1.098–2.440	0.015	0.083			NA
Microvascular invasion (yes versus no)	<0.001	1.886	1.114–3.191	0.018	0.018	1.917	1.053–3.488	0.033
Tumor encapsulation (none versus complete)	0.002	1.323	0.952–1.841	NS	0.087			NA
Tumor differentiation (III/IV versus I/II)	0.018	1.365	0.989–1.884	NS	0.061			NA
TNM stage (I/II versus III)	<0.001			NA	0.001			NA
CD151 density (<50% versus >50%)	0.002	0.698	0.512–0.951	0.023	<0.001	0.555	0.396–0.777	0.001
Integrin β1 density (high versus low)	0.153			NA	0.054			NA
CD151/ Integrin β1 density*	0.002			0.003	<0.001			<0.001
I verse II		1.571	1.091–2.262	0.015		2.028	1.366–3.013	<0.001
I verse III		0.847	0.515–1.392	NS		0.860	0.491–1.506	NS
I verse IV		0.718	0.438–1.174	NS		0.646	0.380–1.096	NS

The Cox proportional hazards regression model was used.

Abbreviations: AFP, alpha-fetoprotein; CI, confidence interval; HBsAg, hepatitis B surface antigen; HR, hazard ratio; NA, not adopted; NS, not significant.

*Patients were classified into four groups according to their CD151 and Integrin β1 density: group I (n = 93) had high expression of CD151 and Integrin β1, group II (n = 47) had high expression of CD151 but low Integrin β1 expression, group III (n = 58) had high expression of Integrin β1 but low CD151 expression and group I V (n = 103) had low expression of CD151 and Integrin β1. Individual variables were first adopted for their prognostic significance by multivariate analysis, and then the combination of CD151 and Integrin β1 was analyzed.

## Discussion

We previously identified tetraspanin CD151 as a key molecule involved in the invasion and metastasis of HCC, most likely through formation of functionally relevant complexes [8], [18], [19]. Here, we used large-scale proteomics screening techniques [29], [30] to develop a map of the extensive protein-protein “interactome” network centered on tetraspanin CD151 in HCC cells.

An important result in this study was that CD151 organized “a tetraspanin web” with several partners, including integrins α6 and β1, c-Met, Moesin, thrombospondin-1 precursor, neuropilin 1, and GIPC PDZ domain containing family, member 1, and served as a molecular facilitator or adaptor for cell migration. Among these molecules, the integrins were considered the most important molecular partners for CD151 [9]. CD151 contains two extracellular loops and two short cytoplasmic tails. The large extracellular loop is necessary and sufficient for mediating the stable interaction of CD151 with the integrin subunit [11], [12]. Previous reports have revealed that CD151 can form a direct and highly stoichiometric lateral complex with integrins α3, α6, α7, β1, and β4, which are able to resist detergents such as digitonin and Triton X-100 [23]. Anti-CD151 antibodies or CD151 mutants can selectively inhibit integrin α3 and α6-dependent cell migration, whereas upregulation of CD151 enhances experimental metastasis of colon carcinoma and fibrosarcoma cells [31]. Mass spectrometry data analysis showed that integrins α3, α6 and β1 serve as molecular partners for CD151. However, integrin α3 could not be identified by Western blot analysis after immunoprecipitation, which is mainly attributed to the extremely low expression of integrin α3 in HCC cells [8], [13]. It has been well documented that the CD151/integrin complex acts as a functional unit for modulation of physiological and pathological processes, including cell migration, neurite outgrowth, and cell morphology [11], [13], [32]. The identified tetraspanin web provided direct evidence for our previous notion that CD151 formed a complex with integrin α6, and induced epithelial–mesenchymal transition in HCC cells [8]. Integrin β1 not only acts as an adhesive receptor for laminins, but also as an intracellular signaling platform that promotes cell migration and invasion [33]. Our results also showed that the integrin β1 subunit often forms structural complexes with the CD151 and integrin α subunit, in line with a previous report [9]. An important partner in the tetraspanin web is c-Met (hepatocyte growth factor receptor). Recent studies have demonstrated the interaction between tetraspanins and c-Met. Tetraspanin CD82 forms a complex with c-Met and inhibits hepatocyte growth factor (HGF)-induced cancer cell migration by inactivation of small GTP-binding proteins in the Rho family, via c-Met adapter proteins [34]. CD82 also complexes with the ganglioside GM2/GM3 heterodimer, and inhibits cell motility through the CD82-c-Met or integrin-c-Met pathways [15]. CD151 can enhance receptor signaling through integrin β4-mediated pathways and control c-Met-dependent neoplastic growth [35]. Our previous data also revealed that CD151 probably interacted with c-Met and had an adverse effect on the prognosis of HCC patients through HGF/c-Met pathways [18]. Importantly, the proteins Moesin, thrombospondin-1 precursor, neuropilin 1, and GIPC PDZ domain containing family, member 1, were observed in the tetraspanin web. Recently, accumulating evidence has demonstrated that these proteins interact with tetraspanins and modulate cell migration. Upregulation of tetraspanin CD81 expression at the NK cell can induce phosphorylation of ezrin/radixin/moesin proteins and lead to NK cell polarization, thereby facilitating NK cell migration towards various chemokines/cytokines [36]. The Ig superfamily proteins, EWIs, act as linkers to connect tetraspanin-associated microdomains to the actin cytoskeleton, to regulate cell motility and polarity via direct interaction with ezrin/radixin/moesin (ERM) proteins [37]. However, the interaction between CD151 and these proteins, and their roles in HCC cell migration remains to be determined. In addition to having purely structural functions as organizers and facilitators, CD151 also regulates various aspects of trafficking and biosynthetic processing of associated receptors as identified in the tetraspanin network, in line with a previous report [38]. Of the 57 proteins identified in Table 1, 47 have not previously been linked to CD151. Regarding these potential CD151 partners, further experiments are needed to confirm their direct association with CD151, to assess the extent of this association, and to determine functional implications.

Another implication about the characterized network centered on CD151, is that it explains the functional complexity of CD151, and provides a clue for uncovering the mystery. Traditionally, overexpression of CD151 facilitates integrins and growth factor receptors signals, activates Rac and Cdc42, and serves as a transmembrane linker between extracellular integrin domains and intracellular cytoskeleton/signaling molecules, leading to the regulation of actin cytoskeleton, cell spreading, tumor cell motility, and metastasis [26]. However, several papers have reported that expression of CD151 has a negative influence on cell migration and invasiveness [25], [26], [27]. The identified tetraspanin web addressed these conflicting results. On one hand, CD151 can form functional complexes with different partners and give rise to different roles in cell adhesion, migration, and metastasis in different cells. On the other hand, the molecular partners in the tetraspanin web play an opposite role in the function of CD151. For example, the low-density lipoprotein receptor precursor, one of the molecular partners identified in this study, was found to counteract the role of CD151 in the progression of HCC (unpublished data). Another important result that arose from our studies is that CD151 played a crucial role in cell migration, invasiveness, and metastasis of HCC in an integrin β1-dependent manner. Overexpression of CD151 has been associated with HCC progression [8], [18], [19]. Recent studies have demonstrated that the altered expression of β1 integrins in HCC, and overexpression of β1 integrins correlate with aggressive phenotypes and metastasis of HCC [7], [39]. However, it is undeniable that HepG2 with very low level of metastatic potential displays high level of β1 integrin, and that a highly positive proportion of β1-integrin expression is observed in most HCC samples [8]. Tetraspanins provide a platform to organize proteins on the cell membrane, which has great flexibility. Each tetraspanin may have specific partners for interaction and can interact with different proteins and in that way contribute to variability, and probably functional specificity, of the complex [40]. Furthermore, the exposure of a CD151 epitope involved in this binding is cell-type specific, and may vary depending on the physiological and pathological status of the tissues involved [21]. We noted that CD151 formed a functional complex with integrin β1 in TEM. We postulated that integrin β1, in concert with associated-CD151 molecules, might support HCC progression, rather than individual integrin β1 molecules. In this study, downregulation of CD151 in HCC cells had no effect on the expression of integrin β1, and vice versa. When either CD151 or integrin β1 was silenced, HCC cells with high expression of CD151/integrin β1 complex displayed markedly impaired motility, invasiveness, and metastasis both *in vitro* and *in vivo*. However, integrin β1-silenced HepG2 cells with low levels of CD151 expression had the same low mobile velocity and weak invasion as that of the parental cell lines. Interestingly, re-expressing CD151 in HepG2 cells reversed the mobile and invasive defects. Moreover, CD151 or integrin β1-slienced HCC cell with high expression of CD151/integrin β1 complex displayed the markedly decreased secretion of MMP9, which was considered as the most important molecule for matrix remodeling [41]. These data indicate that loss of CD151 disrupts integrin β1 association with tetraspanin-enriched microdomains, impairs integrin β1 internalization in cells migrating [31], and modulates the extracellular matrix remodeling through influence of MMP9 secretion [19], [20]. Even more importantly, we identified a role of the CD151/integrin β1 complex in the clinical setting of HCCs. HCC patients with high level of CD151/integrin β1 complex expression had the worst prognosis of all populations examined. A key property of integrins is that they can assume multiple functional states. Integrins on normal cells are mostly inactive and are activated by several stimuli [5]. Our previous study also showed that CD151 could amplify the function of integrin α6 [8]. Therefore, β1 integrins expressed on HCC cells probably harbored various levels of constitutive activity, and at the larger scale, its role in the progression of HCC was activated by the overexpression of CD151.

In conclusion, strong lateral CD151/integrin β1 complex is functionally important, and CD151 serves as a key player in integrin β1-dependent matrix remodeling, cell spreading, and metastasis. Disassociation with CD151-integrin β1 complex is a good choice for developing an anti-metastasis strategy for HCC.

## Materials and Methods

### Cell lines

Human HCC cell lines HCCLM3, MHCC97H, MHCC97L (established in Liver Cancer Institute of Zhongshan Hospital, Fudan University), PLC/PRF/5, Hep3B and HepG2 (American Type Culture Collection, Manassas, VA), and normal hepatocytes L-02 (Chinese Academy of Sciences, Shanghai) were used in this study. Cells were maintained in a 37°C humidified incubator in the presence of 5% CO_2_.

### Immunoisolation of CD151-containing complexes and in-gel tryptic digestion

To identify CD151-associated molecules, immunoprecipitation was performed as previously described [20]. Briefly, 5×10^8^ HCCLM3 cells were lysed in 40 ml lysis buffer containing 10 mM Tris, pH 7.5, 150 mM NaCl, 1 mM CaCl_2_, 1 mM MgCl_2_, 1% Brij 97, and protease inhibitors for 2 h at 4°C. The lysate was centrifuged at 16,000×g for 15 min, and the supernatant was precleared with a combination of protein A/G-agarose beads (Amersham Biosciences, PA USA) precoated with BSA and goat serum (Sigma-Aldrich, MO, USA) for 2 h at 4°C. Then, the lysate was incubated with a specific antibody coupled to protein A/G-agarose beads for 2 h at 4°C. Immobilized immune complexes were then washed four times with lysis buffer, eluted using 1% Triton X-100, and acetone-precipitated. Proteins were resolved by 10% SDS-polyacrylamide gel electrophoresis (PAGE) under non-reducing conditions, and detected by immunoblot analysis using specific antibodies.

Gels were stained with colloidal Coomassie Blue (Bio-Rad, CA USA), excised, and destained. Then, gel pieces were incubated in 100% acetonitrile for 10 min, dried, and incubated in 100 mM ammonium bicarbonate containing 10 mM dithiothreitol (DTT) for 30 min at 56°C. After cooling to room temperature, gel pieces were incubated with 55 mM iodoacetamide in 100 mM ammonium bicarbonate for 20 min in the dark, at room temperature, washed with 100 mM ammonium bicarbonate for 20 min, dehydrated with 100% acetonitrile (ACN), and dried. Then, gel pieces were digested with 100 ng of trypsin and 25 mM ammonium bicarbonate (Roche Applied Science, IN USA) overnight at 37°C. Peptides were extracted successively with 50 µl of 5% formic acid for 15 min, and 100 µl of 100% acetonitrile for another 15 min at 37°C.

### Two-dimensional liquid chromatography coupled with tandem mass spectrometry (2D-LC-MS/MS) and data analysis

Proteins were vacuum-dried and resuspended in 5% ACN containing 0.1% formic acid. LC-MS/MS analyses were performed using a Nano Aquity UPLC system (Waters, Milford, MA) coupled to a LTQ Orbitrap XL mass spectrometer (Thermo Electron, Bremen, Germany), and equipped with an online nanoelectrospray ion source (Michrom Bioresources, Auburn, CA). The capillary column used in this study was a BEH300 C18 reverse phase (75-µm inner diameter, 20 cm). To perform LC-MS/MS analysis, samples were injected into the trap-column at a flow rate of 15 µl/min for 3 min, and subsequently a 3-step linear 105-min gradient (flow rate, 300 nl/min) from 5 to 80% acetonitrile in 0.1% (v/v) formic acid was performed. All data were collected in centroid mode using a data-dependent acquisition mode. After acquisition of a full MS scan (m/z: 400–2000 Da), in the first scan event, the three most intense ions present above a threshold of 105 counts were subsequently isolated for fragmentation (MS/MS scan). The collision energy for the MS/MS scan events was preset at a value of 25%. All MS/MS data were identified using SEQUEST (v.28, Bioworks 3.3 software package, Thermo Electron) against the Human International Protein Index (IPI) database (IPI human v3.45 FASTA with 71983 entries). The search results were checked by examination of the Xcorr (cross-correlation) and the ΔCn (delta normalized correlation) scores. In this study, an Xcorr value of greater than 1.5, 2.0, and 2.5, respectively, for 1, 2, and 3 charged peptides and a ΔCn greater than 0.1 were accepted as positive identification [28]. These peptides were converted into a gene symbol and rearranged according to GO functional enrichment provided by DAVID website. Then, “tetraspanin web” centered on CD151 was organized by the cytoscape (Ver: 2.6.2, http://www.cytoscape.org/), a package to build network, searching target genes that interacted with CD151 and removing the genes that were not contained in our target genes using data source (NCBI Entrez EUtilities Web Service Client).

### Transfection of lentiviral vectors CD151 or integrin β1 shRNA

The pGCSIL-GFP-shRNA-CD151 [8] and pGCSIL-GFP-shRNA-integrin β1 were constructed (pGCSIL, a lentiviral vector, Shanghai GeneChem Co. Ltd); pGCSIL-GFP was used as a negative control. The sequence of shRNA targeting integrin β1 is as follows: 5′-GACTGAGCATGATGAAAGT-3′. The lentiviral vectors and pHelper were co-transfected into 293T cells. Culture supernatants were collected, concentrated, and used as virus stocks. The lentivirus was transfected into HCCLM3 cells with a multiplicity of infection (MOI) of 20–50 (optimal MOI, 30). pcDNA3-CD151cDNA plasmids, kindly provided by Hansoo Lee (Kangwon National University, Korea), were transfected into HepG2 cells using a lentiviral vector as previously described [8].

### Immunocytochemistry (ICC), confocal laser scanning microscopy and flow cytometry

HCCLM3 cells were used to show the co-localization of CD151 and integrin β1. Monoclonal mouse anti-human CD151 (11G5a, 1∶100; Serotec, OX5 1GE, UK) and monoclonal rabbit anti-human integrin β1 antibody (EP1041Y, 1∶250; abCAM, CA USA) were used for fluorescence cytostaining as previously described [8]. The localization of CD151 and integrin β1 was detected by confocal laser scanning microscopy (LSM510; Zeiss, Germany). Anti-human integrin β1/FITC antibody (BD Pharmingen, CA, USA) was used to investigate the expression of integrin β1 in HCCLM3, MHCC97H, MHCC97L, PLC/PRF/5, Hep3B, and HepG2 via flow cytometry using MoFlo (DakoCytomation, Fort Collins, CO) [18]. Isotype-matched immunoglobulin G served as a control. Propidium iodide (1 µg/ml, Sigma-Aldrich, MO, USA) was added to exclude dead cells.

### Immunoblotting and reverse transcription polymerase chain reaction (RT-PCR)

Immunoblotting and RT-PCR were used to detect the expression of CD151 and integrin β1 in HCCLM3, short hairpin RNA (shRNA)-CD151-HCCLM3, HCCLM3-Mock, shRNA-integrin β1-HCCLM3, MHCC97H, MHCC97L, PLC/PRF/5, Hep3B cells, HepG2-CD151 (HepG2-pcDNA3-CD151-cDNA), HepG2-Mock (HepG2-pcDNA3), HepG2-CD151-shRNA-CD151 and HepG2-CD151-shRNA-integrin β1 [19], [42]. Mouse anti-human CD151 monoclonal antibody (11G5a, 1∶200; Serotec, OX5 1GE, UK) and monoclonal rabbit anti-human integrin β1 antibody (EP1041Y, 1∶500; abCAM, CA, USA) were used for immunoblotting, and glyceraldehyde-3-phosphate dehydrogenase (GAPDH, 1∶5000; Chemicon, Billerica, MA, USA) was used as an internal control. Primers of CD151 for RT-PCR analysis: 5′-ACTTCATCCTGCTCCTCATCAT-3′ and 5′-TCCGTGTTCAGCTGCTGGTA-3′; integrin β1: 5′-GCTAAAATGCTGGCACCCTAA-3′ and 5′-ATAGTGCTCCCCAATGAAAGTAGAGA-3′; GAPDH: 5′-GGCATCCTGGGCTACACTGA-3′ and 5′-GTGGTCGTTGAGGGCAATG-3′. The relative expression of messenger RNA (mRNA) was analyzed by the comparative cycle threshold (Ct) method. GAPDH was used as an internal standard. All experiments were performed in triplicate.

### Invasion assay, migration assay, and enzyme-linked immunosorbent assay (ELISA)

Invasive ability of shRNA-CD151-HCCLM3, HCCLM3-Mock, shRNA-integrin β1-HCCLM3, HepG2-CD151, HepG2-Mock, HepG2-CD151-shRNA-CD151 and HepG2-CD151-shRNA-integrin β1 cells were assayed as described elsewhere [19].

Migration ability of shRNA-CD151-HCCLM3, HCCLM3-Mock, shRNA-integrin β1-HCCLM3, HepG2-CD151, HepG2-Mock, HepG2-CD151-shRNA-CD151 and HepG2-CD151-shRNA-integrin β1 were detected as described elsewhere [43].

The concentrations of MMP9 and MMP2 in a conditioned medium from shRNA-CD151-HCCLM3, HCCLM3-Mock, shRNA-integrin β1-HCCLM3, HepG2-CD151, HepG2-Mock, HepG2-CD151-shRNA-CD151 and HepG2-CD151-shRNA-integrin β1 were determined by ELISA with a human MMP9 ELISA kit (R&D Systems) and a human MMP2 ELISA kit (R&D Systems) respectively, essentially as described previously [19]. All experiments were performed in triplicate.

### Metastasis assay

Metastasis nude model of shRNA-CD151-HCCLM3, HCCLM3-Mock, shRNA-integrin β1-HCCLM3, HepG2-CD151, HepG2-Mock cells, HepG2-CD151-shRNA-CD151 and HepG2-CD151-shRNA-integrin β1 was constructed [44]. Serial sections were made for every tissue block from the lung, and the total number of lung metastases was counted under the microscope as described previously [18]. Each group included five animals.

### Patients and follow-up

Tumor specimens taken from areas next to the margin of tumors were obtained from 301 consecutive patients who underwent curative liver resection between 1997 and 2000 in Zhongshan Hospital of Fudan University. Curative resection was defined as complete resection of all tumor nodules and the cut surface being free of cancer by histologic examination; having no cancerous thrombus in the portal vein (main trunk or two major branches), hepatic veins, or bile duct, and having no extrahepatic metastasis [19]. HCC diagnosis was based on the World Health Organization criteria. Tumor differentiation was defined according to the Edmondson grading system [45]. Liver function was assessed by Child-Pugh score system. Tumor staging was determined according to the sixth edition of tumor node metastasis classification of Union Internationale Contre le Cancer. Detailed clinicopathological characteristics were listed in Table 2. Ethical approval was obtained from the research ethics committee of Zhongshan Hospital, and written informed consent was obtained from each patient. Follow-ups were terminated until March, 2007. The median follow-up was 64 months (range, 4–121 months). Follow up procedures were described in our previous study [19]. Treatment modalities after relapse were given according to uniform guidelines as previously described [19].

### Tissue microarray (TMA) and immunohistochemistry

TMA was constructed as described previously [19]. The mouse anti-human CD151 (1∶100, 11G5a, Serotec, OX5 1GE, UK) and monoclonal rabbit anti-human integrin β1 antibody (EP1041Y, 1∶250; abCAM) antibodies were used to detect the expression of CD151 and integrin β1, respectively, based on a two-step protocol as previously described [8]. The density of positive staining of CD151 and integrin β1 was measured as described elsewhere [8].

### Statistical analysis

Statistical analysis was done with SPSS software, version 12.0 (SPSS, Chicago, IL). Overall survival (OS) and time to recurrence were defined as described previously [19]. The cumulative recurrence and OS rates were calculated by the Kaplan-Meier method and the log-rank test. Cox's proportional hazards regression model was used to analyze independent prognostic factors. P<0.05 was set as the level of statistical significance.

## Supporting Information

Figure S1 **Expression of integrin α1, α2, α3, α6 and β1 mRNA in HCCLM3 cells.** Expression of integrin α1, α2, α3, α6 and β1 mRNA in HCCLM3 cells was detected by qRT-PCR.
(TIF)
