# Supplementary figures and images for: Profiling of the Tetraspanin CD151 Web and Conspiracy of CD151/Integrin β1 Complex in the Progression of Hepatocellular Carcinoma
Source: PLoS One. 2011 Sep 22;6(9):e24901. doi: 10.1371/journal.pone.0024901 (PMC3178554; doi:10.1371/journal.pone.0024901)

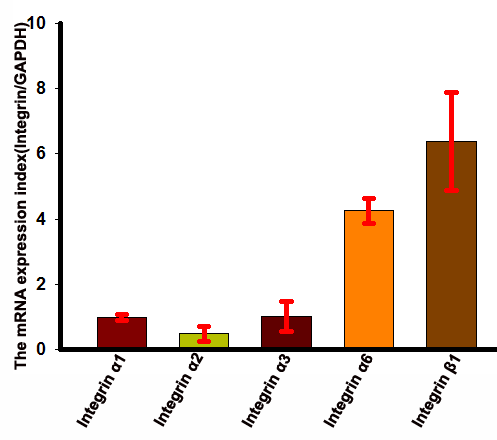

Supplement: Figure S1 — Expression of integrin α1, α2, α3, α6 and β1 mRNA in HCCLM3 cells. Expression of integrin α1, α2, α3, α6 and β1 mRNA in HCCLM3 cells was detected by qRT-PCR. (TIF) [file pone.0024901.s001.tif]
